# Drought-Driven Rhizosphere Microbiome and Metabolome Remodeling in Wild vs. Cultivated *Saccharum arundinaceum*

**DOI:** 10.3390/plants14223407

**Published:** 2025-11-07

**Authors:** Sijie Huang, Haibi Li, Jinju Wei, Hui Zhou, Yanhang Tang, Yiyun Gui, Kai Zhu

**Affiliations:** 1 Guangxi Key Laboratory of Sugarcane Genetic Improvement, Key Laboratory of Sugarcane Biotechnology and Genetic Improvement (Guangxi), Ministry of Agriculture and Rural Affairs, Sugarcane Research Institute, Guangxi Academy of Agricultural Sciences, Nanning 530007, China; huangsijie@gxaas.net (S.H.); lihaibi@gxaas.net (H.L.); jjwei@gxaas.net (J.W.); zhouhui@gxaas.net (H.Z.); tyhasp@gxaas.net (Y.T.); 2 Guangxi South Subtropical Agricultural Science Research Institute, Guangxi Academy of Agricultural Sciences, Chongzuo 532415, China

**Keywords:** rhizospheric soil microbes, metabolites, key functional bacteria, *Saccharum arundinaceum* Retz., drought-resistant

## Abstract

Sugarcane is highly sensitive to the variations in soil moisture content capacity, and upregulated water stress efficiency restricts its development and crop output. Rhizospheric microbes and metabolites play key roles to mitigate the adverse effects of abiotic stresses, i.e., drought stress. The drought-tolerant wild sugarcane relative, *Saccharum arundinaceum* Retz., remains poorly characterized with respect to its rhizosphere microbial community dynamics under water limitation. To address this, we analyzed drought-associated shifts in the rhizosphere microbiome and metabolome by comparing native plants from a long-term arid habitat in Guangxi, China, with plants from an irrigated cultivation environment. We analyzed the effects of agronomic traits, soil properties, enzyme activities, and 16S rRNA sequencing and untargeted metabolomics to characterize microbial communities and metabolites, with correlation analyses. Results demonstrated that wild plants possessed thicker stems, higher proline levels, and increased antioxidant enzyme activity. Their rhizospheres were enriched with *Actinobacteria*, *Proteobacteria*, and *Chloroflexi*, which exhibited upregulated urease and acid phosphatase activities. Metabolites linked to phosphotransferase systems and sugar metabolisms were also more abundant. Positive correlations between these microbes, metabolites, and drought traits reveal site-specific microbial–metabolic modules that confer drought resilience, providing valuable insights for sugarcane breeding programs.

## 1. Introduction

Drought stress, intensified by global climate change, poses a critical threat to plant growth, ecosystem stability, and agricultural productivity worldwide [1,2]. Climate models project increasing frequency and severity of drought events across major agricultural regions, exacerbating water scarcity and reducing soil moisture capacity [3]. Emerging research highlights the pivotal role of plant-associated microbiomes, particularly in the rhizosphere mediating plant drought responses [4,5]. Specific microbial taxa can enhance stress tolerance efficiency by modulating root architecture, producing osmolytes or stress-responsive hormones, and improving water-use efficiency [6]. Nonetheless, drought may also disrupt these plant–microbe interactions, leading to shifts in microbial community composition, and potentially modulate plant development and resilience [7,8]. The fundamental challenge for cultivated sugarcane is that its key domestication traits (high sucrose, thick juicy stems, fast growth) are intrinsically linked to drought sensitivity. The primary agricultural strategy to address these challenges is breeding and genetic introgression. By cross-breeding cultivated sugarcane with its wild relatives like *S. arundinaceum*, plant breeders aim to transfer the resilient “survival traits” (deep roots, high WUE, osmotic adjustment) into high-yielding genetic background, developing advance cultivars that can withstand drought without sacrificing productivity [9,10].

The rhizosphere microbiome, together with root-derived metabolites, plays a pivotal role in modulating plant drought tolerance efficiency. Beneficial microbes, such as plant-growth-promoting rhizobacteria (PGPR) and arbuscular mycorrhizal fungi aid in enhancing water and nutrient uptake, inducing systemic tolerance, and mediating hormonal signaling, such as abscisic acid and auxin modulation [11,12,13]. Under stress conditions, plants adjust their exudation patterns releasing enhanced levels of osmolytes, sugars, amino acids, organic acids, and secondary metabolites, which in turn shape the assembly and activity of microbial communities in the rhizosphere [14,15]. Importantly, metabolites, such as proline, trehalose, antioxidants, and signaling molecules serve as molecular links that modulate microbial recruitment, stress-responsive gene expression, and biochemical interactions between plants and microbes [12,16]. Emerging studies suggest that drought-induced changes in root exudates alter microbial composition, and in turn, the microbiome can modulate host metabolism and stress responses [5,17]. However, these findings underscore the interdependent nature of plant–microbe–metabolite interactions during stress adaptation and upgrade interactive studies to elucidate their functional dynamics.

Despite growing interest, several gaps remain in our understanding of how wild and cultivated plants differentially recruit and interact with rhizosphere microbiomes under drought conditions. The majority of existing research has focused on the model crop species, often overlooking wild relatives that naturally evolved under water-limited environments and may harbor adaptive microbial associations [17]. Comparative studies contrasting rhizosphere microbial shifts between wild and domesticated plants under drought stress are scarce, leaving unsolved story about how domestication has shaped microbial resilience and recruitment [18,19]. These limitations highlight critical need for robust comparative analyses of wild versus cultivated plant systems, leveraging integrative microbiome–metabolome frameworks to explore the functional interplay underlying drought adaptation strategies. Comparative microbiome–metabolome studies showed that crop domestication significantly alters plant’s microbial communities and metabolites it produces, with domesticated maize and sorghum often exhibiting reduced beneficial microbes and increased reliance on fertilizers, alongside shifts in secondary metabolites, such as tannins and benzoxazinoids. Wild relatives typically possess more diverse microbiomes, potentially containing higher abundances of beneficial symbionts and microbes associated in nutrient cycling, while also having different metabolic profiles that contribute to their adverse environmental adaptations [19,20,21].

*Saccharum arundinaceum* Retz., a wild relative of cultivated sugarcane, is notably drought-resilient, providing a valuable model for studying stress-adaptive traits [22,23]. This study investigates how its rhizosphere microbial communities and soil metabolite profiles differ between natural and managed environments. By integrating physiobiochemical analyses with 16S rRNA amplicon sequencing and non-targeted metabolomics, we aim to characterize environment-specific microbial taxa, functional biomarkers, and metabolite–microbe associations under drought stress. Ultimately, our research elucidates the drought-responsive features of the *S. arundinaceum* rhizosphere, offering insights for breeding and management strategies to enhance drought resilience in sugarcane.

## 2. Results

### 2.1. Environmental Variation, Morphological and Biochemical Responses in S. arundinaceum

**Figure 1:**
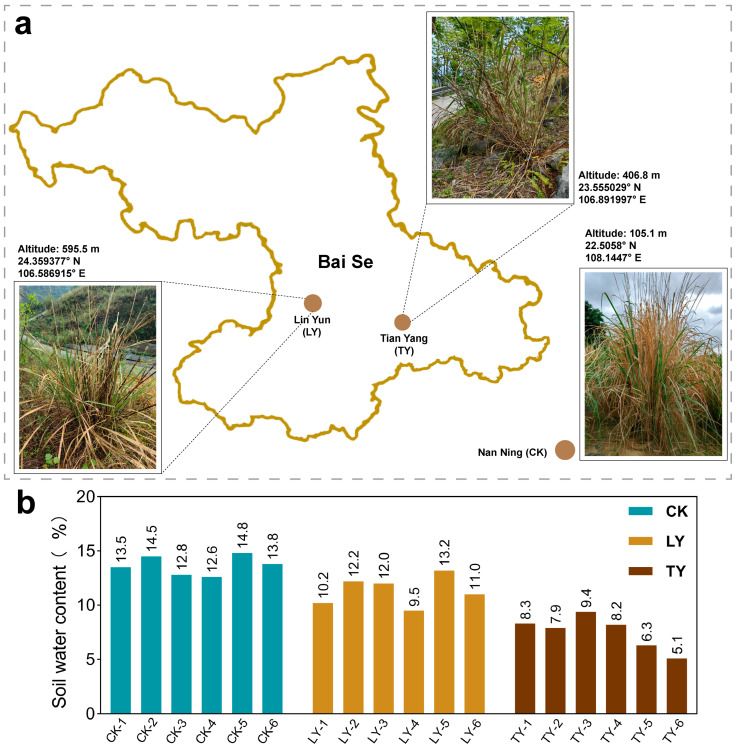
Sampling locations and soil moisture content at experimental sites. Geographical map showing the sampling sites (**a**), and soil moisture content at each site (**b**). CK represents the cultivated sugarcane field, LY and TY denotes wild environment in Lingyun County and Tianyang County, respectively.

To investigate the rhizospheric microbial and metabolic responses of *S. arundinaceum* to contrasting environmental conditions, plant and soil samples were collected from different locations of Guangxi province, China, such as two wild habitats in Baise [Malingtun, Lin Yun (LY), and Qiaoma, Tian Yang (TY)] and cultivated setting at the Sugarcane Research Institute of Guangxi Academy of Agricultural Sciences, Nanning, Guangxi, China (Figure 1a). The two wild sites were characterized by exposed karst topography with thin, rocky soil. LY had not received rainfall for approximately three months prior to sampling, yet *S. arundinaceum* individuals exhibited robust growth with an average height of around 2 m, frequent flowering, and moderate population density in small clusters. In contrast, plants from the TY location showed significant drought stress symptoms, including widespread leaf yellowing and wilting, consistent with the shallow, water-deficient (approx. 7–8% of soil moisture content) and nutrient-poor soil. The cultivated site, by comparison, featured managed growth conditions with regular irrigation and fertilization, providing a stark contrast to the wild drought-prone environment.

**Figure 2:**
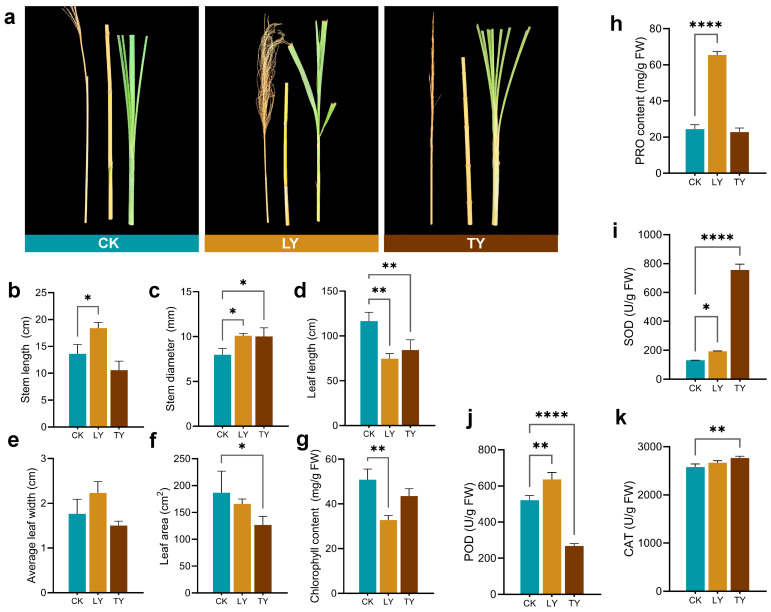
Plant phenotypes and physio–biochemical parameters of *S. arundinaceum* from different sites. Representative growth phenotypes of *S. arundinaceum* from each sampling site (**a**), morphological traits, i.e., stem length, stem diameter, leaf length, average leaf width, and leaf area (**b**–**f**), and physiological and biochemical indicators, such as chlorophyll content, proline content, and enzyme activities of superoxide dismutase (SOD), peroxidase (POD), and catalase (CAT) (**g**–**k**).Statistical analyses were performed using Student’s *t*-tests for pairwise comparisons and one-way ANOVA for multiple group comparisons. Significance levels are indicated as *p* < 0.05 (*), *p* < 0.01 (**), and *p* < 0.001 (****).

According to site characterization, assess the morphological and physiological traits of *S. arundinaceum* collected from the cultivated control (CK), and two wild drought-prone sites (LY and TY) (Figure 2a). Compared to CK, plants from wild environments developed significantly thicker stems (1.27-fold in LY and 1.26-fold in TY, Figure 2c), suggesting adaptive structural reinforcement under water-limited conditions. However, leaf development was generally suppressed in wild plants, as indicated by shorter leaf length (0.64-fold in LY and 0.72-fold in TY; both statistically significant, Figure 2d), smaller leaf area (0.89-fold in LY and 0.68-fold in TY; significant in TY, Figure 2f), and reduced chlorophyll content (0.65-fold in LY and 0.86-fold in TY; significant in LY, Figure 2g).

Stress-responsive physiological traits further supported drought exposure in wild habitats. As shown in Figure 2h–k, in LY plants, proline content (2.68-fold), POD (1.22-fold), and SOD activities (1.45-fold) were significantly upregulated relative to CK, whereas CAT activity remained unchanged. Interestingly, TY plants exhibited a contrasting antioxidant profile, with significantly downregulated POD activity (0.52-fold) and dramatically higher SOD activity (5.70-fold), reflecting possible site-specific stress response mechanisms.

### 2.2. Soil Physicochemical Properties

The environmental context plays a key role in shaping plant and microbial responses. We analyzed the physicochemical properties of rhizospheric soil collected from the different sites. Due to the relatively uniform soil surface conditions in the cultivated region (CK), three composite soil samples were prepared by mixing three subsamples. In contrast, the wild habitats (LY and TY) exhibited considerable heterogeneity; thus, soil was collected from the three distinct locations within each site, and three subsamples per location were pooled to generate representative samples.

Significant differences (*p* < 0.05) were observed in available phosphorus, total nitrogen, and organic matter content (OMC) among the different sites. Soil pH ranged from slightly acidic to mildly alkaline across sampling areas. The rhizosphere soil in CK, TY1, and TY2 were slightly acidic, while TY3, LY1, LY2, and LY3 exhibited weak alkalinity. CK soil showed the highest available phosphorus content (1958.67 ± 188.31 mg/kg), whereas LY2 exhibited the lowest levels, contributing to the overall lower phosphorus content in the LY site (113.33 ± 31.77 mg/kg). In contrast, total nitrogen levels were significantly higher in wild soil, with LY and TY displaying minimum values of 256.15 ± 9.4 g/kg, markedly above the CK average (82.5 ± 0.14 g/kg). Organic matter content also varied significantly among samples. CK soil had the lowest value (1383.9 ± 48.05 g/kg), while TY soil exhibited the highest OMC (8995.23–15,025.97 g/kg), and LY soil ranged from 4110.50 to 5399.60 g/kg (Table S1).

### 2.3. Soil Enzyme Activity

**Figure 3:**
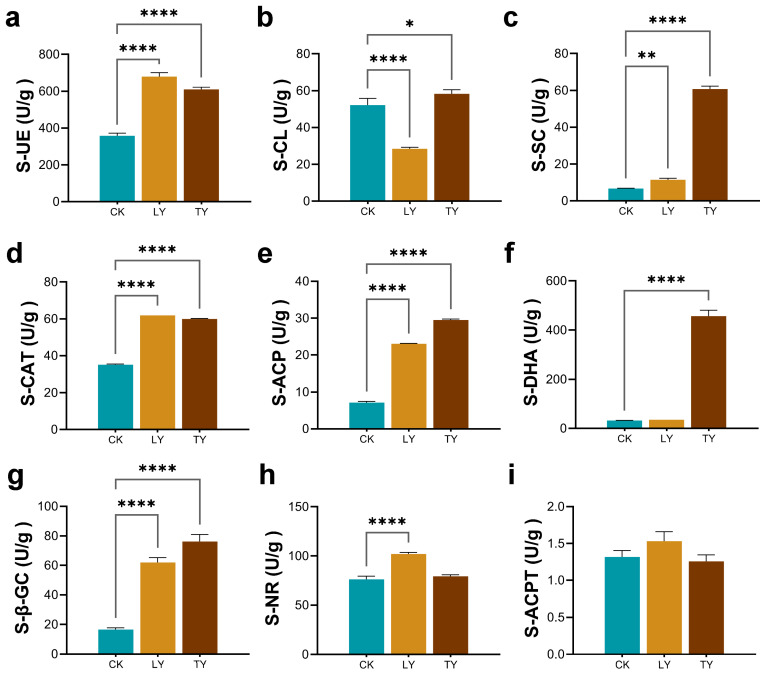
Activities of soil enzymes in rhizospheric samples from different environments. (**a**) Urease (S-UE); (**b**) Cellulase (S-CL); (**c**) Sucrase (S-SC); (**d**) Catalase (S-CAT); (**e**) Acid phosphatase (S-ACP); (**f**) Dehydrogenase (S-DHA); (**g**) β-Glucosidase (S-β-GC); (**h**) Nitrate reductase (S-NR); and (**i**) Acid protease (S-ACPT). Each data point represents an individual sample from distinct locations within a region. Statistical analyses were performed using Student’s *t*-tests for pairwise comparisons and one-way ANOVA for multiple group comparisons. Significance levels are indicated as *p* < 0.05 (*), *p* < 0.01 (**), and *p* < 0.001 (****).

Different habitat conditions altered soil enzyme activities, which reflect microbial functional capacity and nutrient cycling dynamics (Figure 3a–i). Compared to the cultivated site (CK), both wild sites (LY and TY) exhibited significantly higher urease (S-UE) activity, with LY (679.26 U/g) and TY (609.60 U/g) markedly exceeding CK (357.33 U/g). Soil sucrase (S-SC) activity showed divergent patterns, such as LY showed reduced activity (28.49 U/g) relative to CK (52.20 U/g), whereas TY showed significant increase (58.30 U/g), indicating site-specific differences in microbial carbohydrate metabolism. Cellulase (S-CL) activity was significantly higher in both wild environment, especially in TY (60.77 U/g), suggesting accelerated cellulose decomposition and carbon turnover under drought stress. Catalase (S-CAT) activity was also upregulated in LY (61.85 U/g) and TY (59.91 U/g) compared to CK (35.21 U/g), reflecting stronger oxidative stress responses in microbial communities under arid conditions.

Phosphorus cycling-related enzyme acid phosphatase (S-ACP) was significantly upregulated in LY (23.05 U/g) and TY (29.53 U/g) compared to CK (7.12 U/g), indicating enhanced microbial efforts to mobilize phosphorus in nutrient-deficient soil. Notably, only TY exhibited dramatically elevated dehydrogenase (S-DHA) activity (457.02 U/g), relative to CK (32.37 U/g), indicating higher microbial metabolic intensity. Similarly, β-glucosidase (S-β-GC) activity was significantly higher in LY (61.83 U/g) and TY (76.11 U/g) than CK (16.54 U/g), underscoring the increased capacity for polysaccharide breakdown in drought-exposed soil. Nitrate reductase (S-NR) activity was significantly enhanced in LY (101.90 U/g) but not in TY, suggesting localized intensification of nitrogen assimilation pathways. In contrast, no significant differences were observed in acid protease (S-ACPT) activity across the different sites. These enzyme activity profiles collectively suggest that drought-associated wild environment harbor rhizosphere microbial communities with enhanced functional potential for nutrient acquisition, organic matter turnover, and oxidative stress tolerance, laying the foundation for subsequent microbial diversity and community composition analysis.

### 2.4. Soil Microbiota Composition Analysis of S. arundinaceum Rhizospheric Soil in Different Regions

This study employed 16S rRNA amplicon sequencing to analyze the bacterial communities in nine composite rhizospheric soil samples. The samples were sourced from three distinct environments, i.e., cultivated control site (CK, Sugarcane Research Institute) and two wild, drought-affected regions (T1-LY and T2-TY). Sequencing produced a total of 554,432 high-quality sequences, with a per-sample yield of 35,296 to 68,178 reads. Clustering at 97% sequence similarity threshold resulted in the identification of 8697 operational taxonomic units (OTUs). Subsequent taxonomic annotation of OTUs representative sequences was performed to assess the microbial composition across samples. Diversity analysis revealed distinct microbial community structures across the different regions. Unique OTUs were defined by individual sampling locations within each site. 1524, 1784, and 1783 unique OTUs were found in CK, T1, and T2, respectively, with 1792 OTUs shared among all samples. Alternatively, when site-level consensus OTUs were used (i.e., OTUs present in all three subsamples per site), CK, T1, and T2 contained 442, 121, and 219 unique OTUs, respectively, with 690 OTUs shared across all groups.

**Figure 4:**
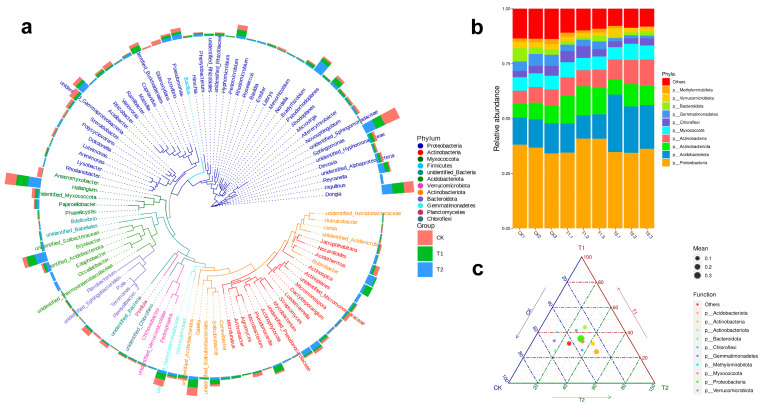
Taxonomic annotation based on OTUs analysis. Phylogenetic tree of microbial genera constructed from OTUs. Branch and sector colors indicate corresponding phyla, while the stacked bar plots on the outer ring represent genus-level abundance across different samples (**a**), stacked bar chart showing the relative abundance of microbial taxonomy with phyla (**b**), and ternary plot illustrating the distribution of major microbial taxa across the three sampling groups (**c**).

A phylogenetic tree constructed from the top 100 most abundant genera (Figure 4a) highlighted major taxonomic divergences among samples. At the phylum level, relative abundance analyses (Figure 4b) showed that *Proteobacteria*, *Acidobacteriota*, and *Actinobacteriota* dominated the rhizosphere microbiome across all regions. To visualize compositional preferences and identify group-specific dominant taxa, ternary plots were generated using the top 10 taxa at each taxonomic level (Phylum to Species) based on the average abundance (Figure 4c). These plots illustrated clear distributional biases among microbial clades. Most taxa were broadly distributed, indicating compositional heterogeneity in response to environmental conditions. Notably, *Proteobacteria* formed large nodes on the plot, confirming its role as a core and abundant group in the rhizosphere of *S. arundinaceum*. In contrast, low-abundance but environmentally responsive phyla, including *Methylomirabilota*, *Bacteroidota*, and *Gemmatimonadetes*, appeared more associated with the moist, nutrient-rich soil of the cultivated CK group.

**Figure 5:**
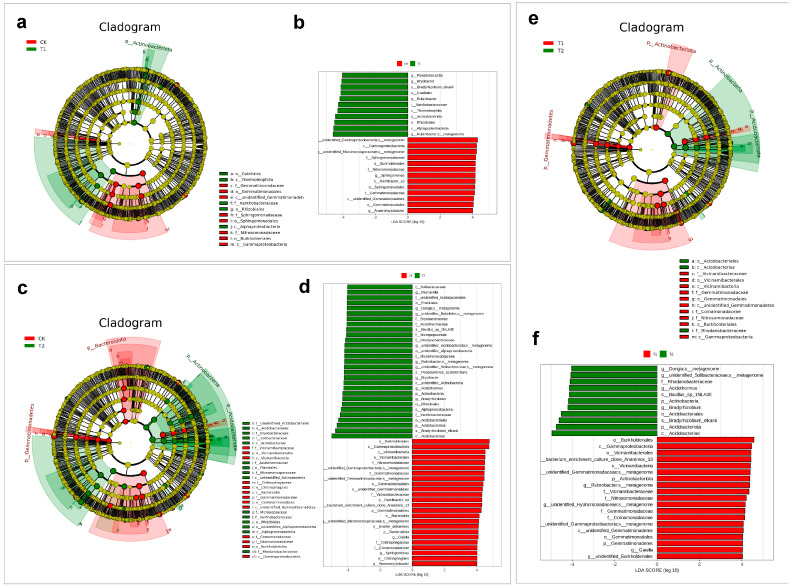
Comparative taxonomic profiles of rhizospheric soil across different regions. Phylogenetic cladogram comparing CK and LY groups. Concentric rings represent hierarchical taxonomic ranks from phylum to genus (or species). Each node corresponds to a taxon, with node size proportional to its relative abundance. Yellow nodes indicate taxa with no significant differences between groups; red and green nodes represent taxa significantly enriched in the red- and green-colored groups, respectively, and identified as potential biomarkers (**a**), histogram of LDA scores (LDA > 4) for differentially abundant taxa between CK and LY, indicating statistically significant biomarkers (**b**), phylogenetic cladogram, LDA score distribution for CK vs. TY comparisons (**c**,**d**), and phylogenetic cladogram, LDA score distribution for LY vs. TY comparisons, respectively (**e**,**f**).

Comparative taxonomic analyses were conducted to elucidate compositional differences in rhizospheric microbial communities among the three regions (Figure 5). Phylogenetic cladograms (Figure 5a,c,e) were constructed to visualize the hierarchical distribution of microbial taxa from phylum to genus (or species), with circle size reflecting relative abundance. Differential biomarkers were identified using LEfSe analysis, where taxa with LDA scores > 4 were considered statistically significant (Figure 5b,d,f). In the CK vs. T1 (LY) comparison, f_Gemmatimonadales, f_Sphingomonadaceae, and o_Burkholderiales (within c_Gammaproteobacteria) were enriched in the cultivated (CK) group, whereas o_Gaiellales, c_Thermoleophilia, f_Xanthobacteraceae, o_Rhizobiales, and c_Alphaproteobacteria were predominant in the T1 group. These findings were corroborated by LDA score bar plots, which provided intuitive visualization of group-specific biomarkers. Additionally, SIMPER (Similarity Percentage) analyses was applied to quantify the contribution of individual OTUs to community dissimilarities between groups. The top ten taxa contributing to Bray–Curtis dissimilarity are presented in bubble plots, where bubble size reflects relative abundance and the *x*-axis denotes the percentage contribution of each taxon. These detailed results are provided in the Supplementary Materials (Figure S1).

In the CK vs. T2 (TY) comparison, biomarkers, such as f_Vicinamibacteraceae, o_Vicinamibacterales, and f_Chitinophagaceae were enriched in CK, while T2 harbored higher abundances of f_unidentified_Acidobacteriales, o_Acidobacteriales, and f_Bryobacteraceae. For the T1 vs. T2 comparison, T1 was characterized by enhanced levels of f_Vicinamibacteraceae, f_Gemmatimonadaceae, f_Comamonadaceae, f_Nitrosomonadaceae, o_Burkholderiales, and c_Gammaproteobacteria, whereas T2 was enriched in o_Acidobacteriales, c_Acidobacteriae, and f_Rhodanobacteraceae. These results highlight the distinct microbial signatures associated with wild drought-prone versus cultivated environment, reflecting adaptive shifts in rhizosphere microbiota in response to environmental diversity.

### 2.5. Shifts in Microbial Potential Under Drought and Cultivation

**Figure 6:**
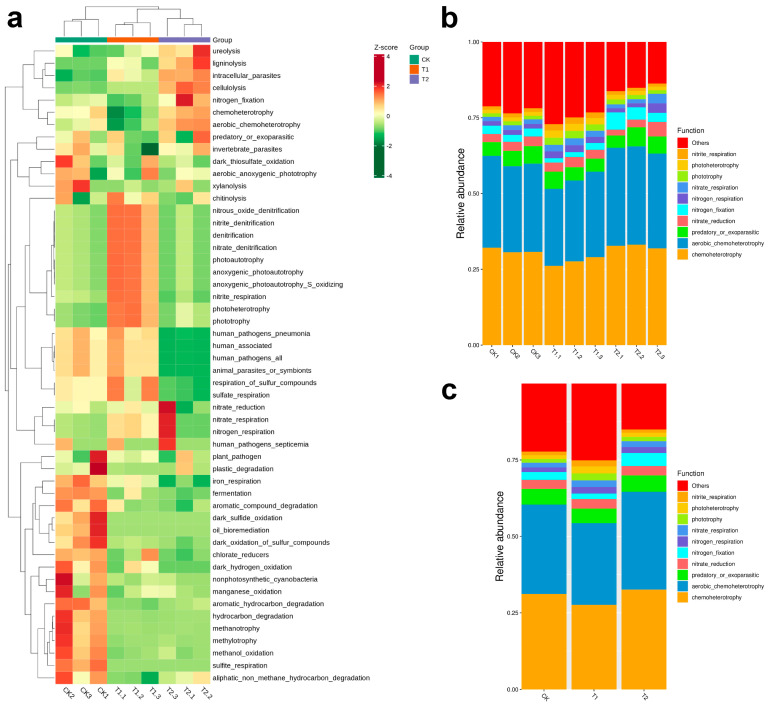
Functional prediction and annotation of bacterial communities. Heatmap showing hierarchical clustering of the relative abundances of predicted functional annotations (**a**), Bar plot of the relative abundances of FAPROTAX-predicted functions based on OTU-level annotation for each sample (**b**), and Bar plot showing the group-level relative abundances of FAPROTAX-predicted functions based on the OTUs (**c**).

The ecological functions of microbial communities in different rhizospheric environments, functional prediction and annotation were performed using FAPROTAX based on OTUs profiles (Figure 6a–c). The clustering heatmap (Figure 6a) revealed distinct functional profiles among the different groups (CK, T1, and T2), indicating that environmental conditions significantly shaped microbial functional capacities. Notably, functions associated with nitrogen cycling, such as “nitrate reduction” and “denitrification” were more enriched in the T1 group, suggesting enhanced activity of denitrifying microbes under stressed wild conditions. In contrast, the CK group showed higher relative abundance of “aerobic anoxygenic phototrophy,” to specific microbial adaptations to oxygen and light conditions of the cultivated environment. The T2 group exhibited distinct enrichment in sulfur-related functions, particularly “sulfate respiration,” highlighting potential alterations in sulfur metabolism under its respective soil conditions. Additionally, functions related to human pathogens and pollutant degradation varied across groups, reflecting diverse ecological roles and potential impacts on soil health. Collectively, these findings indicated that drought and control cultivation exert significant influence not only on microbial community structure but also on their functional potential, with implications for biogeochemical cycling and rhizosphere resilience.

### 2.6. Non-Targeted Metabolomic Analysis of Rhizospheric Soil

**Figure 7:**
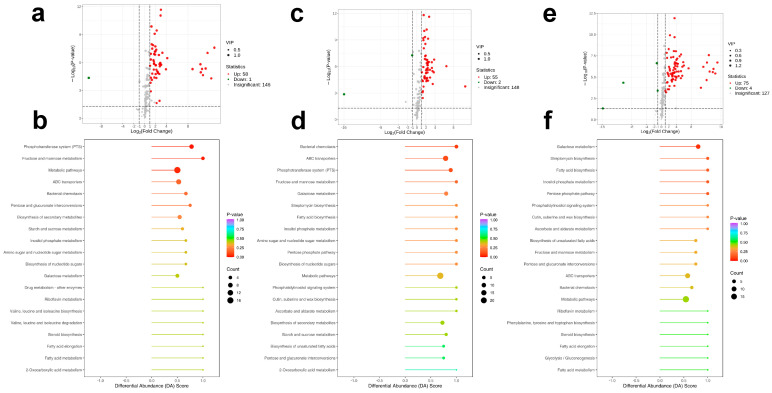
Non-targeted metabolomic analysis of soil samples. Volcano plot of differentially expressed metabolites between CK and LY (**a**), abundance score plot of metabolites between CK and LY (**b**), Volcano plot of differentially expressed metabolites between CK and TY (**c**), abundance score plot of metabolites between CK and TY (**d**), Volcano plot of differentially expressed metabolites between LY and TY (**e**), and abundance score plot of metabolites between LY and TY (**f**).

Changes in rhizospheric microbial communities are often accompanied by alterations in soil metabolic profiles, which provide functional insights into microbial–environment interactions. To further elucidate the metabolic consequences of shifts in rhizospheric microbial communities across different environments, non-targeted soil metabolomic profiling was performed based on the composite soil samples from six sites per region (CK, LY, and TY) (Figure 7a–f). In the CK vs. LY comparison, volcano plot analysis revealed 155 significantly differential features, with 58 upregulated and only 1 downregulated in LY, suggesting an overall increase in metabolite abundance under wild drought conditions (Figure 7a). Pathway enrichment analysis (Figure 7b) indicated that the phosphotransferase system (PTS) and fructose and mannose metabolism were significantly enriched, highlighting functional differentiation in transport and carbohydrate metabolic pathways. These differences may reflect microbial adaptations to nutrient availability and environmental stress in the LY rhizosphere.

In the CK vs. TY comparison, 206 differential features were identified, including 75 upregulated and 4 downregulated in TY (Figure 7c), suggesting elevated metabolic activity in the wild drought-prone soil. Enriched pathways included bacterial chemotaxis, ABC transporters, and phosphotransferase system (PTS) (Figure 7d), indicating distinct microbial strategies for motility, nutrient acquisition, and carbon metabolism potentially linked to the rhizosphere adaptation of *S. arundinaceum* in TY. Similarly, the LY vs. TY comparison yielded 205 differential features, with 55 upregulated and 2 downregulated in TY (Figure 7e). Key enriched pathways included galactose metabolism, streptomycin biosynthesis, and fatty acid biosynthesis (Figure 7f), suggesting functional divergence in secondary metabolism and membrane lipid synthesis. These findings point to regional differences in microbial metabolic strategies and further underscore the influence of environmental conditions on rhizosphere functional potential. Collectively, these results demonstrated that the drought and control cultivation not only shape the microbial community composition but also restructure the metabolic landscape of rhizosphere soil, laying the foundation for integrative microbiome–metabolome analyses.

### 2.7. Correlations Among Soil Metabolites and Soil Microbial Communities

**Figure 8:**
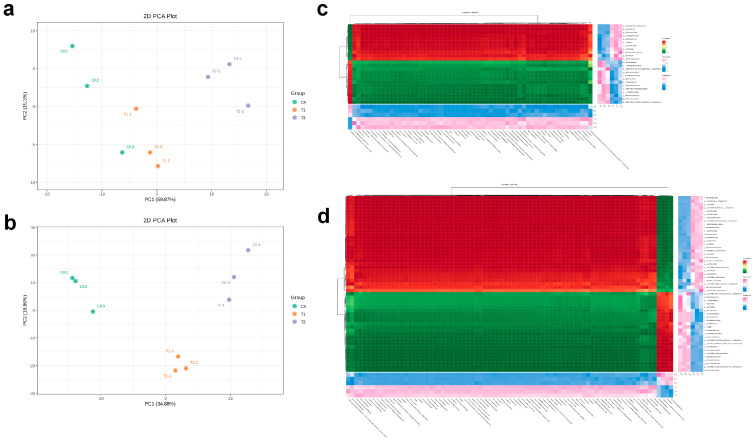
Integrative analysis of soil metabolome and microbiome. Principal component analysis (PCA) of differential soil metabolites (**a**), PCA of differential microbial communities (**b**), Hierarchical clustering heatmap of Pearson correlations between differential metabolites and microbial taxa in CK vs. LY (**c**), and Hierarchical clustering heatmap of Pearson correlations between differential metabolites and microbial taxa in CK vs. TY (**d**).

The functional relationships among drought-responsive soil metabolites and rhizospheric microbial communities, integrated microbiome–metabolome correlation analyses were conducted. Principal component analysis (PCA) based on significantly altered metabolites and microbial taxa showed clear separation among CK, LY, and TY groups, confirming distinct site-specific compositional profiles (Figure 8a,b). Pearson correlation-based hierarchical clustering heatmaps were constructed to explore the ecological interactions between differential microbes and metabolites. In the CK vs. LY comparison (Figure 8c), drought-enriched taxa, such as Rubrobacter, Ramlibacter, and Sphingomonas exhibited strong positive correlations with stress-associated metabolites, including allantoin, 4-hydroxyproline, and specific glycerophospholipids, suggesting enhanced microbial functions in osmoprotection and membrane stabilization under drought conditions [24]. Under drought conditions, microbes enhance osmoprotection and membrane stabilization by altering their glycerophospholipid composition, with some evidence suggesting increased amounts of negatively charged intact polar lipids (IPLs) and the accumulation of specific compounds like phosphatidylcholine (PC). Microbes remodel their cell membranes by increasing IPLs, which have bilayer-stabilizing properties, and can also synthesize protective substances and modulate membrane structure to prevent phase transitions and maintain cell integrity [25]. Conversely, negatively correlated taxa, including members of *Thermoleophilia* and *Acidobacteria*, may represent microbial groups suppressed or outcompeted under water-limited stress.

Similarly, the CK vs. TY analysis (Figure 8d) revealed a distinct set of microbial–metabolite associations. Taxa, such as Arthrobacter, Gaiella, and unclassified Gemmatimonadaceae showed strong positive correlations with sulfur-containing and phenolic compounds, potentially linked to microbial sulfur metabolism and antioxidant activity in the TY rhizosphere. Notably, members of Nocardioidaceae and Frankiales displayed consistent negative correlations with a wide range of metabolites, reflecting selective microbial recruitment and metabolic reprogramming in response to environmental stresses at the TY site. These findings highlight that drought-associated abiotic stress restructure the microbiome–metabolome interaction network in the location-specific manner. The observed co-variations between particular microbial taxa and key drought-responsive metabolites underscore the integral role of rhizosphere microbiota in modulating chemical environments and contributing to the adaptive capacity of *S. arundinaceum* under wild and cultivated conditions.

### 2.8. Correlation Analysis Between Drought Resistance-Related Indexes of S. arundinaceum and Differential Microbial Abundance in Rhizosphere Soil

**Figure 9:**
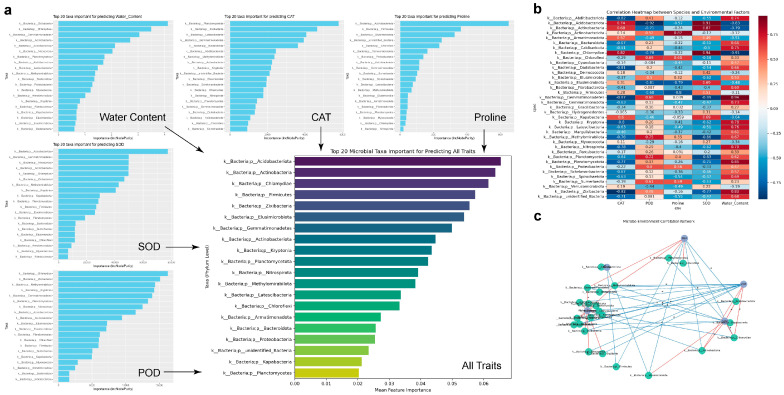
Correlation analysis between drought-related traits of *S. arundinaceum* Retz. and the abundance of differential rhizospheric microbes. Univariate and multivariate analysis of the relationships between soil water content, *S. arundinaceum* Retz. SOD, CAT, POD activities, proline content, and the abundance of differential microbes across regions, based on multiple random forest models (**a**), Heatmap showing correlations between phylum-level differential rhizosphere microbial abundance and drought-related physiological traits of *S. arundinaceum* Retz. (**b**), and Network diagram illustrating correlations between phylum-level differential rhizosphere microbial abundance and drought-related traits of *S. arundinaceum* Retz. (**c**).

Stress-responsive physiological parameters of plants are associated to shifts in rhizospheric microbial communities are critical for unraveling plant–microbe interactions under environmental stress. To further elucidate the associations between drought-resistance-related traits of *S. arundinaceum* and the structure of its rhizospheric microbial community, we employed an integrated approach combining random forest modeling (with cross-validation to ensure model robustness) and Pearson correlation analysis as an independent validation of the predictions (Figure 9). The random forest models identified key microbial phyla contributing to variation in individual drought-related parameters, including soil water content, activities of SOD, CAT, and POD, and proline concentration as well as in the combined multivariate model (Figure 9a). Notably, *Actinobacteria*, *Proteobacteria*, and *Chloroflexi* consistently emerged as top predictors across multiple models, suggesting their prominent roles in shaping the rhizosphere under drought stress conditions. Pearson correlation heatmap (Figure 9b) revealed positive associations between these microbial groups and physiological indicators, such as antioxidant enzyme activities and osmoprotectants, particularly CAT, POD, and proline, serving as an independent check to corroborate the predictive outcomes of the random forest models. Furthermore, the correlation network (Figure 9c) highlighted tightly connected microbial clusters that co-varied with drought-responsive traits, implying coordinated microbial responses to environmental stressors. Collectively, these results reveal a coherent pattern of association between specific microbial taxa and drought-related physiological adaptations, underscoring the integrative role of the rhizosphere microbiome in enhancing the drought resilience of *S. arundinaceum*.

## 3. Discussion

Drought triggers significant restructuring of the rhizosphere microbiome, with the response varying considerably between plant genotypes. Wild-type plants maintain higher abundance of documented drought-resilient and plant-growth-promoting rhizobacteria, particularly from the Actinobacteria and Proteobacteria phyla [26,27]. In contrast, the microbiome of cultivated varieties shifts more drastically, recruiting a greater proportion of neutral or potentially pathogenic taxa. In *S. arundinaceum*, these drought-induced shifts were characterized by enhanced microbial diversity and the enrichment of Actinobacteria, Proteobacteria, and Chloroflexi in wild genotypes. This microbial restructuring was coupled with elevated physiological drought adaptations, including antioxidant enzyme activity (CAT, POD), osmolyte (proline) accumulation, and the enrichment of key metabolite pathways, such as the phosphotransferase system, sugar metabolism, and sulfur respiration [28,29]. Integrative analyses confirmed strong associations between specific microbial phyla and physiological indicators of drought tolerance, revealing site-specific microbial–metabolite modules that potentially underpin resilience in wild *S. arundinaceum*. Wild species serve as a genetic reservoir, contributing essential genes for stress tolerance, yield, and disease resistance to cultivated sugarcane [30].

The enrichment of *Actinobacteria*, *Proteobacteria*, and *Chloroflexi* under drought aligns with observations in other drought-adapted systems. For example, *Proteobacteria* and *Actinobacteria* were enriched in arid squash rhizospheres and associated with osmotic stress genes [3,31], and in millet systems where carbohydrate-metabolism pathways and phosphorus cycling genes were upregulated [32]. Similarly, random forest and correlation models in our study identified these phyla as key predictors of drought-resilience indices, reinforcing their ecological significance. Metabolomic differences, especially in fructose/mannose metabolism, PTS, and sulfur cycling mirror findings in other drought-tolerant cereals where sugar transport, secondary metabolism, and stress metabolite networks underpin adaptation [5,33]. Importantly, the integration of microbial and metabolomic data expands beyond standard taxonomic surveys. Few studies in sugarcane relatives have combined OTUs-level microbiome sequencing and non-targeted soil metabolomics across wild vs. cultivated contexts. This dual-omics approach allowed us to detect specific microbe–metabolite associations, suggesting a feedback loop in which drought alters root exudate profiles that recruit beneficial taxa, which in turn modulate metabolite dynamics and plant physiology consistent with growing evidence in model crops [34,35].

The comparative analyses reveals that plants in wild, unmanaged environments maintain more diverse and potentially robust rhizosphere microbiomes under drought than cultivated counterparts pattern observed in other wild relatives of domesticated crops [36]. This microbial resilience may stem from long-term co-evolution with variable water availability, as opposed to the homogenization of microbial communities in managed agricultural soil. The differing metabolomic and microbial assemblies suggest that domestication and cultivation practices alter plant–microbe adaptation strategies, warranting attention to wild germplasm and environmental context in breeding programs [37]. Functionally, the strong co-variation between microbial taxa (*Actinobacteria*, *Proteobacteria*, *Chloroflexi*) and drought-linked traits (high proline, SOD/POD/CAT activity) in combined analysis implies that specific taxa are integral to maintaining rhizosphere homeostasis under stress conditions. Elevated urease activity in wild soil suggests enhanced nitrogen mineralization capacity, likely driven by increased microbial adaptation to nutrient-poor, drought-prone conditions. This supports the growing consensus that microbiome composition influences plant physiological resilience beyond nutrient acquisition [38,39,40].

Further, enriched pathways identified by heatmap and gradient analysis particularly sulfur metabolism and carbohydrate transport suggest these microbial communities are adapting to stress by altering their nutrient processing, particularly in managing energy and nutrient exchange for survival. This highlights microbial functional plasticity, where adaptive responses to stress include enhanced capabilities in sulfur oxidation or reduction and flexible carbohydrate transport and metabolism to maintain cellular function [41]. These findings should be validated in future field studies across different soil types and incorporate shotgun metagenomics, metatranscriptomics, or metaproteomics to confirm microbial functional potential and actively expressed pathways. This is necessary to confirm their efficacy within the heightened complexity of natural microbial communities.

## 4. Materials and Methods

### 4.1. Soil Sampling and Characterization

Rhizospheric soil samples of *Saccharum arundinaceum* were collected from the three different field sites in Guangxi province, China, selected to represent contrasting moisture regimes and environment. The field experiment locations are as Lingyun County (LY, 595.5 m elevation, 24.359377° N, 106.586915° E) of a drought-prone rocky hillslope with semi-natural vegetation and no rainfall for approximately three months prior to sampling, Tianyang County (TY, 406.8 m elevation, 23.555029° N, 106.891997° E) of a steep karst terrain featuring shallow, nutrient-poor soil interspersed with rock crevices, where measured soil moisture was nearly 7–8%. When the soil moisture content (SMC) is less than 15%, it is considered as the drought level [42]. The cultivated sugarcane nursery at Guangxi Academy of Agricultural Sciences, Nanning (CK, ~105 m elevation, 22.5058° N, 108.1447° E) of managed agricultural site under regular irrigation and cultivation approaches.

Soil samples at each site were collected from six independent plants (*n* = 6) and treated separately. Rhizosphere soil was obtained by carefully excavating plants, manually removing large stones and roots, and gently shaking to collect closely adhered soil. The soil was homogenized and passed through a sterile 2 mm sieve, as recommended for improved microbial and metabolomic consistency. Four aliquots (~10 g each) were placed into sterile 50 mL centrifuge tubes, immediately flash-frozen in liquid nitrogen in the field, and subsequently stored at −80 °C for metabolomic profiling, microbial community, enzyme assay analysis, respectively. Remaining bulk soil was air-dried at room temperature, stored in paper envelopes, and used for the determination of soil physico-chemical parameters, such as moisture content, pH, organic matter, total nitrogen, available phosphorus, and electrical conductivity using standard protocols.

### 4.2. Measurement of Plant Phenotypic Traits, Physiological Parameters, and Antioxidant Enzyme Activities

Morphological characteristics, i.e., stem diameter, leaf length, leaf width, and leaf-area expansion were measured on photosynthetically fully mature leaves and corresponding internode sections collected in the field. Stem diameter was recorded using digital calipers, while leaf area was calculated as leaf length × maximum leaf width × 0.75, following established methods for grass species. Chlorophyll content was estimated non-destructively using a portable SPAD-502 chlorophyll meter (Konica Minolta, Tokyo,Japan), with three readings per leaf averaged for each plant. For physiological and biochemical assays, leaf tissue (~0.5 g) was ground in liquid nitrogen and extracted in ice-cold phosphate buffer (50 mM, pH 7.0). Proline content was determined by the acid–ninhydrin colorimetric method, with absorbance measured at 520 nm and proline quantity calculated from the standard curve. Antioxidant enzyme activities were assayed by spectrophotometrically. Superoxide dismutase (SOD) activity was assessed by its inhibition of nitroblue tetrazolium (NBT) photoreduction with absorbance recorded at 560 nm (one unit defined as 50% inhibition). Peroxidase (POD) activity was measured via guaiacol oxidation in presence of H_2_O_2_ at 470 nm, and catalase (CAT) activity by monitoring H_2_O_2_ decomposition at 240 nm [43]. All enzyme assays were conducted at 25 °C with three biological replicates (*n* = 3) per sample and normalized per gram fresh weight of leaf tissue.

### 4.3. Soil Physicochemical Properties and Enzyme Activity Assays

Soil physicochemical properties were analyzed using air-dried samples (*n* = 3) passed through a 2 mm sieve. Soil pH was determined in 1:2.5 (*w*/*v*) soil-to-water suspension with a glass electrode. Moisture content was measured gravimetrically after drying at 105 °C. Organic matter was quantified using the Walkley–Black method [44], total nitrogen via Kjeldahl digestion, and available phosphorus using either Olsen or Bray-I extraction, depending on soil pH. Fresh rhizospheric soil (5–10 g) were used for enzyme assays after 2 mm sieving. Urease (S-UE), acid phosphatase (S-ACP), dehydrogenase (S-DHA), and catalase (S-CAT) activities were measured using standard colorimetric protocols. Sucrase (S-SC), cellulase (S-CL), β-glucosidase (S-β-GC), and nitrate reductase (S-NR) activities were determined spectrophotometrically according to established methods. All enzyme activities were expressed per gram of dry soil and conducted in triplicate using fresh composites stored at −80 °C to preserve activity.

### 4.4. Microbial Community Analysis

Total genomic DNA was extracted from rhizospheric soil using the MagicPure Stool and Soil Genomic DNA Kit (TransGen, Beijing, China). DNA quality and concentration were assessed by agarose gel electrophoresis, and extracted DNA was diluted to 1 ng/μL with sterile water for downstream applications. PCR amplification targeted specific regions using barcoded primers and Phusion^®^ High-Fidelity PCR Master Mix with GC Buffer (New England Biolabs, MA, USA) to ensure high amplification efficiency and fidelity. The primer sets targeted the 16S rRNA gene V4 region (515F/806R) for bacterial diversity and the 18S rRNA gene V4 region (528F/706R) for eukaryotic microbial diversity [45,46]. ITS amplification for fungal diversity was initially performed but is not included in the analyses due to low coverage and limited interpretability [47]. Similarity percentage (SIMPER) analysis was conducted using the simper function in the vegan package of R (v4.3.1) to identify taxa contributing most to community dissimilarities among treatments. Linear discriminant analysis effect size (LEfSe) was applied to detect differentially abundant taxa with consistent biological relevance. The LEfSe analysis was performed in R using the microbiomeMarker package, with an LDA score threshold of 2.0 and *p* < 0.05 as the significance criterion.

PCR products were verified on 2% agarose gels, purified using magnetic bead-based cleanup, and quantified by spectrophotometry. Equimolar amounts of bacterial and eukaryotic amplicons were pooled, and the pooled library was further validated by gel electrophoresis and purified using a Qiagen gel extraction kit (Thermo Fisher Scientific, MA, USA). Sequencing libraries were prepared using the TruSeq^®^ DNA PCR-Free Sample Preparation Kit (Illumina, CA, USA), and library quality was assessed by Qubit fluorometry and qPCR. Qualified libraries were sequenced on the Illumina NovaSeq 6000 platform, generating paired-end reads of 250 bp. Raw reads were demultiplexed based on barcode and primer sequences, and barcodes/primers were trimmed. Paired-end reads were merged using FLASH v1.2.11 to obtain raw tags, which were subjected to quality filtering in QIIME v1.9.1, including truncation at the first low-quality base (Q ≤ 19 for ≥3 consecutive bases), removal of sequences shorter than 75% of the expected length, and chimera detection with VSEARCH. After filtering, effective tags were obtained for OTU clustering at 97% similarity. Sequencing depth ranged from 35,296 to 68,178 high-quality reads per sample, with a total of 554,432 sequences across all samples, providing sufficient coverage for downstream bacterial and eukaryotic microbial community diversity analyses.

### 4.5. Soil Untargeted Metabolomics Analysis

Fresh soil samples were collected (n = 6), weighed, flash-frozen in liquid nitrogen, and stored at −80 °C until analysis. Samples were freeze-dried and ground to a fine powder at room temperature. Approximately 0.5 g of soil powder was extracted with 1 mL methanol:isopropanol:water (3:3:2, *v*/*v*/*v*), vortexed for 3 min, and ultrasonicated for 20 min. Extracts were centrifuged at 12,000 rpm (4 °C) for 3 min. Supernatants were collected, spiked with 20 μL internal standard (10 μg/mL), and evaporated under nitrogen. Residues were freeze-dried for derivatization. Derivatization involved incubation with 100 μL methoxyamine hydrochloride in pyridine (15 mg/mL) at 37 °C (2 h), followed by addition of 100 μL BSTFA with 1% TMCS and further incubation at 37 °C (30 min). 200 μL aliquot was diluted to 1 mL with n-hexane, filtered through 0.22 μm syringe filter, and stored at −20 °C prior to GC-MS analysis within 24 h. Metabolite profiling was performed on an Agilent 8890 GC coupled to 5977B MS with DB-5MS column (30 m × 0.25 mm i.d., 0.25 μm film). Helium was used as carrier gas at 1.2 mL/min. One microliter samples were injected in split mode (5:1). The oven was held at 40 °C (1 min), ramped to 100 °C at 20 °C/min, then 300 °C at 15 °C/min, and held at 300 °C (5 min). Ion source and transfer line temperatures were set at 230 and 280 °C, respectively. Data were acquired in full scan mode.

Random forest (RF) analysis was performed in Python (v3.10) using the scikit-learn library to identify key OTUs distinguishing among the CK, T1, and T2 treatments. The normalized OTU abundance matrix served as the input, and treatment groups were used as categorical responses. Each RF model included 500 trees (n_estimators = 500) with the Gini impurity criterion and sqrt(p) features considered at each split. Model performance was validated by 10-fold cross-validation, and feature importance was ranked by the mean reduction in Gini index. OTUs consistently within the top 5% importance across comparisons were defined as discriminant taxa. Visualization and interpretation were conducted using matplotlib, seaborn, and SHAP to illustrate variable contributions and model robustness.

### 4.6. Statistical Analysis

Graphs were generated using GraphPad software (v10.6.0). Statistical analyses were performed with SPSS (IBM SPSS, version 27.0). Independent sample *t*-tests, one-way ANOVA, and two-way ANOVA were conducted with significance threshold of *p* < 0.05. Principal component analysis (PCA), partial least squares discriminant analysis (PLS-DA), volcano plots, and heatmaps were carried out using RStudio (v4.4.1). Functional analysis was based on the KEGG database.

## 5. Conclusions and Future Research Directions

This multi-omics study reveals divergent drought resilience strategies in wild and cultivated *S. arundinaceum*. We demonstrate that the wild genotype’s better tolerance is not solely intrinsic but is critically mediated by its enhanced capacity to recruit a protective, drought-responsive rhizosphere microbiome. Isolation of core taxa or metabolites identified here could inform targeted microbial inoculants or soil amendments. Research findings illuminate environment-specific microbial–metabolite networks that underpin drought adaptation in *S. arundinaceum*. Our multi-omics framework bridges compositional and functional dimensions, offering mechanistic insights and potential translational pathways to enhance crop resilience in the era of climate change. This research would be a major step forward for our understanding of *S. arundinaceum*. FAPROTAX predicts functional profiles based on the identity of 16S rRNA sequences and their association with cultured representatives in a database. It does not measure actual gene expression (via metatranscriptomics) or protein abundance (via metaproteomics). A drought-induced shift in a bacterial genus known for “nitrogen fixation” does not confirm that nitrogen fixation genes were expressed or active under the experimental conditions. Through its robust integration of multi-omics methodologies, the study delivers mechanistic validation of the ‘cry for help’ hypothesis applied to plant domestication. Consequently, it argues that the conservation of wild genetic resources is imperative, as they possess not only beneficial plant genes but also a critical capacity to manage microbial genes essential for adaptation to climate change.

## Data Availability

All data generated or analyzed in this study are included in this article. The raw sequencing data were submitted to the NCBI database with the Project ID: PRJNA1321565.

## Supplementary Materials

The following supporting information can be downloaded at: https://www.mdpi.com/article/10.3390/plants14223407/s1, Table S1: Physicochemical properties of rhizospheric soil collected from wild and cultivated environment. Figure S1: SIMPER analysis of OTU-based contributions to differences between CK and LY (a), CK and TY (b), and LY and TY (c). Bubble size denotes the relative abundance of each taxon, and the *x*-axis represents each taxon’s contribution to dissimilarity between groups.
