# Supplementary material for: Drought-Driven Rhizosphere Microbiome and Metabolome Remodeling in Wild vs. Cultivated Saccharum arundinaceum
Source: Plants (Basel). 2025 Nov 7;14(22):3407. doi: 10.3390/plants14223407 (PMC12656359; doi:10.3390/plants14223407)
Supplement: Supplementary file 1 [file plants-14-03407-s001.zip › plants-3895396-supplementary.pdf]

Drought-Driven Rhizosphere Microbiome and Metabolome Remodeling in Wild vs. Cultivated *Saccharum arundinaceum*

Sijie Huang[1,†], Haibi Li[1,2,†], Jinju Wei[1], Hui Zhou[1], Yanhang Tang[1], Yiyun Gui[1,**], Kai Zhu[1,*]

[1]Guangxi Key Laboratory of Sugarcane Genetic Improvement, Key Laboratory of Sugarcane Biotechnology and Genetic Improvement (Guangxi), Ministry of Agriculture and Rural Affairs, Sugarcane Research Institute, Guangxi Academy of Agricultural Sciences, Nanning, 530007, Guangxi, China; emails: sijie_H1994@163.com (SH), jjwei@gxaas.net (JW), zhouhui@gxaas.net (HZ), tyhasp@126.com (YT)

[2]Guangxi South Subtropical Agricultural Science Research Institute, Guangxi Academy of Agricultural Sciences, Chongzuo, 532415, Guangxi, China; email: lihaibi@gxaas.net

Correspondence: zhukai@gxaas.net (K.Z.), guiyiyun218@163.com (Y.G.**)

†These authors contributed equally to this work.

Table S1. Physicochemical properties of rhizospheric soil collected from wild and cultivated environment.

Group	pH	Electric conductivity (μs/cm)	Available phosphorus (mg/kg)	Total nitrogen (g/kg)	Organic matter (g/kg)
CK	6.82	266.0	1958.67±188.31[a]	82.50±0.14[a]	1383.90±48.05[a]
TY1	5.64	199.3	250.00±40.15[b]	406.35±2.33[b]	10707.80±463.78[b]
TY2	6.53	221.0	320.67±65.03[b]	496.40±4.53[c]	15025.97±574.01[c]
TY3	7.10	232.0	260.00±102.43[b]	440.05±0.92[d]	8995.23±74.04[d]
LY1	7.05	186.2	153.33±20.53[b]	259.00±0.50[e]	5137.17±126.11[e]
LY2	7.28	178.0	113.33±31.77[b]	297.95±1.63[f]	5399.60±70.49[ef]
LY3	7.20	59.4	122.67±42.44[b]	256.15±9.40[eg]	4110.50±33.38[f]

Note: CK- cultivated control, TY and LY- wild drought-prone sites

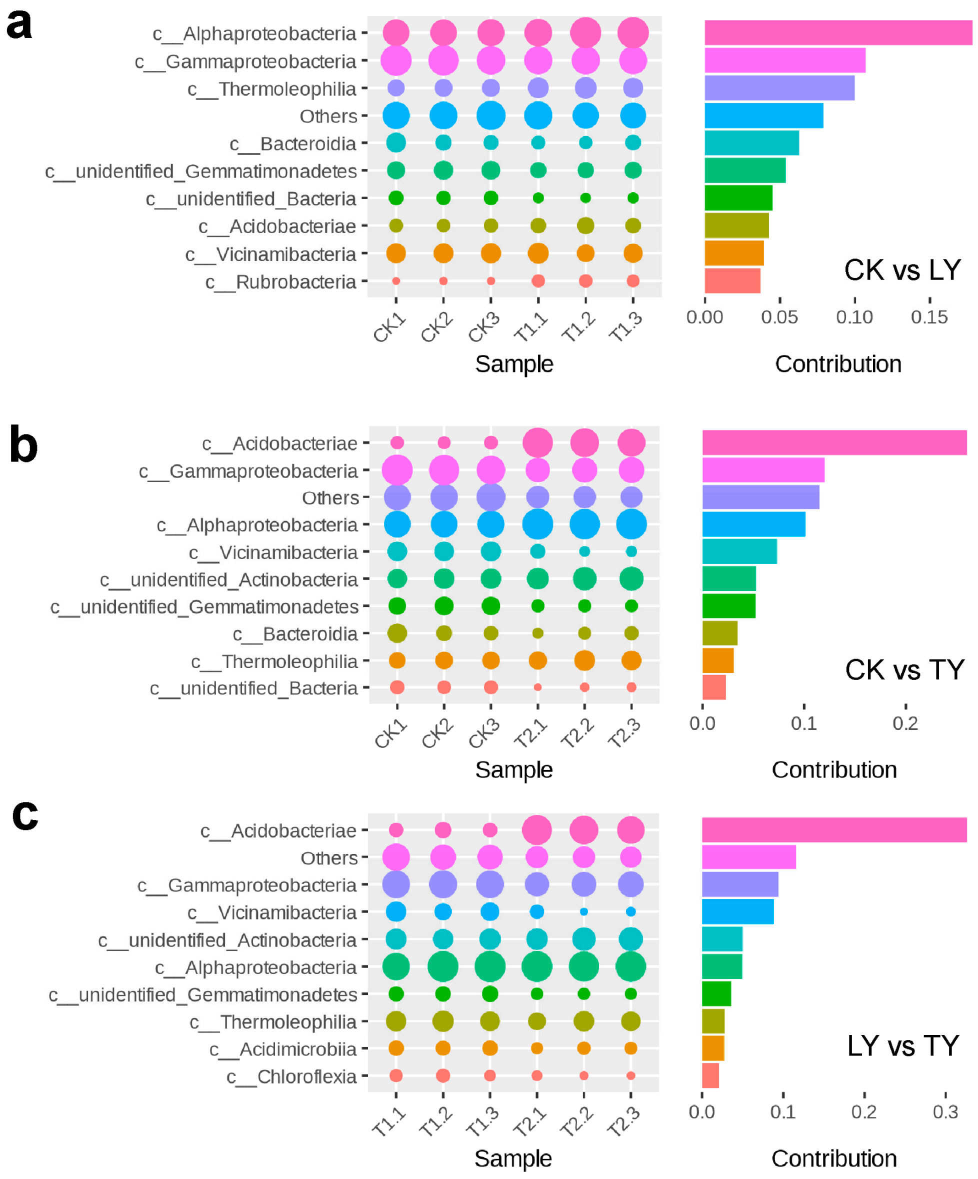

Figure S1. SIMPER analysis of OTU-based contributions to differences between CK and LY (a), CK and TY (b), and LY and TY (c). Bubble size denotes the relative abundance of each taxon, and the x-axis represents each taxon's contribution to dissimilarity between groups.
